# Effects of Cooking and Processing Methods on Phenolic Contents and Antioxidant and Anti-Proliferative Activities of Broccoli Florets

**DOI:** 10.3390/antiox10050641

**Published:** 2021-04-22

**Authors:** Hee Young Kim, Meran Keshawa Ediriweera, Kyung-Hwan Boo, Chang Sook Kim, Somi Kim Cho

**Affiliations:** 1 Interdisciplinary Graduate Program in Advanced Convergence Technology and Science, Jeju National University, Jeju 63243, Korea; hi.khyoung@gmail.com; 2 Subtropical/Tropical Organism Gene Bank, Jeju National University, Jeju 63243, Korea; mk.ediriweera@gmail.com (M.K.E.); khboo@jejunu.ac.kr (K.-H.B.); 3 Department of Biotechnology, College of Applied Life Sciences, Jeju National University, Jeju 63243, Korea; cskim1225@jejunu.ac.kr

**Keywords:** antioxidant capacity, broccoli extracts, processing, radical scavenging

## Abstract

We investigated the effects of cooking (steaming and microwaving) and processing (freeze-drying and hot-air-drying) methods on the antioxidant activity of broccoli florets. 2,2-diphenyl-1-picrylhydrazyl (DPPH^•^), 2,2’-azino-bis(3-ethylbenzothiazoline-6-sulfonic acid) (ABTS^•^), and alkyl^•^ free radical scavenging assays were employed to assess anti-oxidant potentials. The cytoprotective effect against oxidative damage induced by H_2_O_2_ was studied using hepatocellular carcinoma (HepG2) cells. Anti-proliferative effects were assessed in MCF-7 and MDA-MB-231 breast cancer cells. L-sulforaphane in broccoli extracts was quantified using high-performance liquid chromatography (HPLC). Steam and microwave treatments caused increases in total polyphenol content (TPC), whereas the total flavonoid content (TFC) decreased following steam treatment. A slight increase in TFC was observed in the microwaved samples. Extracts of all broccoli samples showed almost identical radical scavenging and cytoprotective effects. HPLC demonstrated that steamed (3 min)-freeze-dried (F-S3) and microwaved (2 min)-freeze-dried (F-M2) samples exhibited elevated levels of L-sulforaphane. In addition, the F-S3 and F-M2 extracts displayed strong anti-proliferative effects in MCF-7 cells, which correlated with L-sulforaphane content. As we observed no significant decrease in the antioxidant activity of broccoli florets, the cooking and processing methods and conditions studied here are recommended for broccoli.

## 1. Introduction

Among cruciferous vegetables, broccoli (*Brassica oleracea* var. *italica*) has been identified as a “super-food” due to its wide range of health benefits [1]. China, India, and the United States are the largest broccoli producers in the world [2]. Broccoli is a rich source of essential minerals, amino acids, dietary fiber, and various phytochemicals [3]. Glucosinolates, isothiocyanates, carotenoids, flavonoids, phenolics, xanthophylls, and sterols are the major phytochemical types found in broccoli [3]. Glucosinolates undergo enzymolysis (by myrosinase) to produce sulforaphanes, which have a wide range of biological activities including antioxidant, anti-inflammatory, and anti-cancer effects [4]. Several epidemiological studies have identified an inverse correlation between the consumption of broccoli and the risk of certain ailments including cancer, cardiovascular diseases, neurological diseases, and diabetes [5].

Although some cruciferous vegetables, including broccoli, can be eaten fresh, they are mainly consumed after cooking. Steaming and microwaving are two widely used household cooking methods [6]. Temperature can affect the levels and bioavailability of health-promoting bioactive components, as well as the physical characteristics and antioxidant contents of foods due to damage, release, or generation of new metabolites [6]. Steaming creates a uniform heat distribution, increasing water retention [7]. On the other hand, microwave heating results in non-uniform heat development and the rapid movement of water toward the surface, affecting the palatability of food [8].

Due to the high perishability of broccoli, its preservation through proper processing methods is crucial. Spoilage can significantly affect the amount of health-promoting bioactive components and their bioavailability, and thus must be avoided [9]. Domestic and industrial preservation methods generally include heat treatment, which can alter the levels of bioactive components and their bioavailability in *Brassica* vegetables, including broccoli [10]. Dehydration is the main principle of many food processing methods. The main aim of dehydration is to minimize microbial spoilage and the rate of deterioration by lowering the water content of food [11]. The water content of food has been reported to affect multiple spoilage pathways through direct roles as a reactant or product [11]. Freeze-drying is one of the most successful food preservation techniques for perishable food items. During freeze-drying, water is removed from food material through the sublimation of ice [12]. Freeze-drying has been reported to maintain the appearance, shape, flavor, and biological activities of food items, making it a promising drying technique [12].

The present investigation explored the effects of various cooking (steaming and microwaving) and processing (air drying and freeze-drying) methods on the antioxidant activity, effects on breast cancer cell proliferation, and hydrogen peroxide (H_2_O_2_)-induced reactive oxygen species (ROS) production of broccoli florets.

## 2. Materials and Methods

### 2.1. Plant Materials

Fresh broccoli (*Brassica oleracea* var. *italica*) was purchased from local markets on Jeju Island, South Korea in 2020. Broccoli florets were manually removed using a clean sharp knife, washed with tap water, and dried on paper towels.

### 2.2. Cooking Processes

After drying on paper towels, broccoli florets were mixed well, and then 5200 g samples were taken and divided into four portions (1300 g per portion). One portion (1300 g) was kept raw and others were cooked using two different methods in triplicate, as described below.

#### 2.2.1. Steaming

A sample of 1300 g of broccoli florets was steamed using a steam vessel with 200 mL of water for 3 min.

#### 2.2.2. Microwave Cooking

A sample of 1300 g of broccoli florets was placed in a glass dish with 100 mL of water. The dishes were covered with cooking bags with holes and cooked in a commercial microwave oven for 2 min at 100–120 °C (230 V-50 Hz AC, 700 W). Another sample of 1300 g of broccoli florets was cooked in the same microwave oven for 4 min under the same conditions.

### 2.3. Drying Experiments

Steamed, microwaved (for 2 and 4 min), and fresh broccoli florets were subjected to both vacuum freeze-drying and hot-air-drying processes, as described below.

#### 2.3.1. Vacuum Freeze-Drying

Steamed, microwaved, and raw broccoli florets (650 g) were vacuum freeze-dried (LP 20; Ilshinbiobase, Dongducheon, Korea) for 2 days and stored in airtight plastic bags.

#### 2.3.2. Hot-Air-Drying

**Figure 1:**
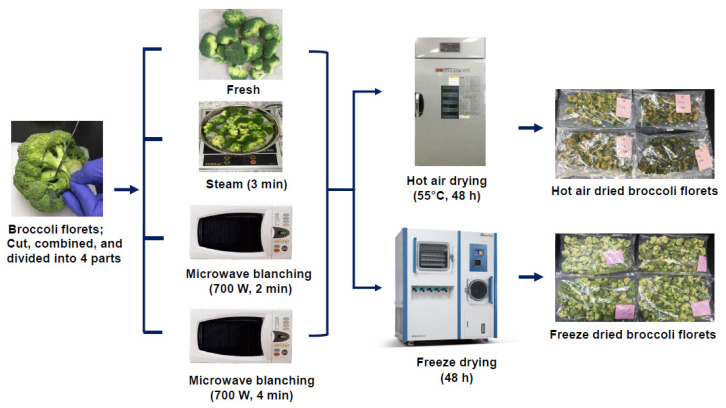
Flow diagram of the cooking and processing methods used for broccoli in the present study.

Broccoli florets were spread evenly on a tray, placed in the drying chamber of a hot-air dryer, and dried at 55 °C for 48 h. The cooking and drying procedures employed in the present study are shown as a flow chart in Figure 1.

### 2.4. Extraction of Plant Material

Following vacuum freeze-drying and hot-air-drying, 10 g of each powdered broccoli sample was extracted into 500 mL of 80% methanol (three times) with sonication for 45 min at room temperature. The resulting extracts were evaporated using a rotary evaporator and stored at −20 °C until used. The extracts were named and coded as follows: fresh-hot-air-dried (H-F), steamed (3 min)-hot-air-dried (H-S3), microwaved (2 min)-hot-air-dried (H-M2), microwaved (4 min)-hot-air-dried (H-M4), fresh-freeze-dried (F-F), steamed (3 min)-freeze-dried (F-S3), microwaved (2 min)-freeze-dried (F-M2), and microwaved (4 min)-freeze-dried (F-M4).

### 2.5. Determination of Total Polyphenol (TPC) and Flavonoid (TFC) Contents

The total polyphenol content (TPC) was determined as previously described [13]. Briefly, 1.375 mL of water and 125 μL of an individual extract were mixed with 500 μL of Folin–Ciocalteu phenol reagent and incubated for 5 min. After 5 min of incubation, 1 mL of 10% Na_2_CO_3_ was added to the reaction mixture, which was incubated at room temperature for a further 30 min in the dark. Following incubation, the absorbance was recorded using a microplate reader. TPC was expressed as milligrams of gallic acid equivalent (GAE) per gram of extract. To assess the TFC contents of the extracts, we followed a previously-described method [13]. Briefly, 40 μL of an extract was mixed with 80 μL of distilled water and 6 μL of 5% NaNO_2_. After 5 min of incubation, 12 μL of 10% AlCl_3_ was mixed with the reaction mixture, which was incubated at room temperature for 6 min. Following incubation, 40 μL of 1 N NaOH was added to the reaction mixture and its absorbance was measured at 510 nm using a microplate reader. TFC was expressed as milligrams of rutin equivalent (RE) per gram of extract.

### 2.6. Analysis of Antioxidant Activity

#### 2.6.1. 2,2-Diphenyl-1-picrylhydrazyl (DPPH) Radical Scavenging Activity Based on Electron Spin Resonance (ESR)

The DPPH radical scavenging activity of the samples was estimated using ESR as previously described with slight modifications [14]. Reaction mixtures were prepared by mixing 30 µL of an extract with 30 µL of freshly prepared DPPH solution (60 μM in ethanol solution). Following mixing, they were incubated at room temperature for 2 min and transferred to Teflon™ capillary tubes using a syringe. ESR spectra were recorded using the following parameters: frequency, 9.43 GHz; power, 5 mW; sweep width, 10 mT; sweep time, 30 s; time constant, 0.03 s; amplitude, 500; and modulation width, 0.8 mT. A magnetic ESR standard (Mn^2+^ marker) was used for comparison of signal intensities, and results were expressed as relative height ratios. Catechin was used as the positive control in this experiment. The percentage radical scavenging activity of each extract was calculated using the following formula: (Absorbance of the control group—absorbance treated group) ÷ Absorbance control group) × 100%. Following percentage radical scavenging activity calculations, EC_50_ values for each extract were generated using GraphPad Prism 7.0 software.

#### 2.6.2. Alkyl Radical Scavenging Activity Based on ESR

Prior to analysis, reaction mixtures were prepared by mixing 20 µL of distilled water, 20 µL of extract, 20 µL of 40 mM (-(4-pyridyl-1-oxide)-N-tert -butylnitrone (4-POBN), and 20 µL of 40 mM 2,2’-Azobis(2-amidinopropane) dihydrochloride (AAPH). Then, the reaction mixtures were incubated at 37 °C for 30 min and transferred to Teflon™ capillary tubes using a syringe. A JES-FA200 ESR spectrometer (JEOL, Tokyo, Japan) was utilized to measure alkyl radical scavenging activity at the Bio-Health Materials Core-Facility in Jeju National University. The following parameter settings were used to record ESR spectra: frequency, 9.43 GHz; power, 7 mW; sweep width, 10 mT; sweep time, 30 s; time constant, 0.03 s; amplitude, 500; and modulation width, 0.2 mT. A magnetic ESR standard (Mn^2+^ marker) was used for comparison of signal intensities and results were expressed as relative height ratios. Catechin was used as the positive control in this experiment. The percentage radical scavenging activity of each extract was calculated using the following formula: (Absorbance of the control group − absorbance treated group) ÷ Absorbance control group) × 100%. Following percentage radical scavenging activity calculations, EC_50_ values for each extract were generated using GraphPad Prism 7.0 software.

#### 2.6.3. 2,2’-Azino-bis(3-ethylbenzothiazoline-6-sulfonic Acid) (ABTS) Radical Scavenging Activity Assay

The ABTS radical scavenging activity assay was performed as previously described with slight modifications [14]. Prior to the assay, fresh ABTS radical solution (7 mM ABTS in 2.45 mM potassium persulfate) was prepared and incubated for 20 h at room temperature. Prepared ABTS stock solution was then diluted with distilled water to obtain an absorbance of 0.700 ± 0.005 at 734 nm. Then, 100 µL of each broccoli extract was mixed with 900 µL of ABTS solution (diluted) and incubated for 2 min. The ABTS radical scavenging activity was measured using the recorded absorbance values, with α-tocopherol as the positive control. The percentage radical scavenging activity of each extract was calculated using the following formula: (Absorbance of the control group—absorbance treated group) ÷ Absorbance control group) × 100%. Following percentage radical scavenging activity calculations, EC_50_ values for each extract were generated using GraphPad Prism 7.0 software.

### 2.7. Cell Culture

Human triple-negative breast cancer (MDA-MB-231), estrogen receptor-positive (MCF-7), and normal mammary epithelial cells (MCF-10A) were cultured using the cell culture media recommended by the American Type Culture Collection (ATCC) and maintained at 37 °C in an atmosphere of 5% CO_2_. The hepatocellular carcinoma (HepG2) cell line obtained from KCLB (Korean Cell line Bank) was cultured in RPMI1640 (Roswell Park Memorial Institute Medium) and maintained at 37 °C in an atmosphere of 5% CO_2_.

### 2.8. 3-(4,5-Dimethylthiazol-2-yl)-2,5-diphenyltetrazolium Bromide (MTT) Assay

The MTT assay was conducted as previously described [15]. Briefly, MDA-MB-231, MCF-7 and MCF-10A cells (5000 cells/well) were seeded in 96-well plates and incubated for 24 h. Then, the cells were exposed to various extracts for 48 h. Following 48 h of incubation, the cells were washed with phosphate-buffered saline (PBS) and incubated with MTT solution (20 μL at 1 mg/mL) for 4 h at 37 °C. Then, 200 μL of dimethyl sulfoxide (DMSO) was added to each well, and the plates were shaken for 45 min on a plate shaker. The absorbance of each well was measured at 570 nm using a microplate reader, and the percentage of cell viability was calculated as described in our recent study. The percentage of cell viability was calculated using the formula (Absorbance of the control group − absorbance treated group) ÷ Absorbance control group) × 100%. The percentage of cell viability values was then used to calculate IC_50_ for each extract using GraphPad Prism 7.0 software (La Jolla, CA, USA).

### 2.9. Measurement of Intracellular ROS Generation

The probe 2′,7′-dichlorodihydrofluorescein diacetate (H_2_DCFDA) was used to measure intracellular ROS production. Briefly, HepG2 cells (40,000 cells/well) were cultured in 96-well plates and incubated for 24 h, pre-treated with broccoli extracts for 6 h, and then exposed to H_2_O_2_ (final concentration, 300 µM) for 10 min to induce ROS formation. Cells were then incubated with H_2_DCFDA for 10 min. Following incubation, fluorescence intensity was measured using a microplate reader at excitation and emission wavelengths of 525 and 475 nm, respectively.

### 2.10. Quantification of L-Sulforaphane via High-Performance Liquid Chromatography (HPLC)

Prior to quantification using HPLC, each broccoli sample (F-F, F-S3, F-M2, F-M4, H-F, H-S3, H-M2, and H-M4) was subjected to small-scale extraction using distilled water and dichloromethane. Briefly, 1 g of each broccoli sample was mixed with 4 mL of distilled water and vortexed for 10 min at room temperature. After resting for 10 min, 15 mL of dichloromethane was added, and the mixture was vortexed for 15 min. The resulting extracts were filtered, evaporated, and subjected to HPLC analysis. The HPLC system was equipped with an ultraviolet detector (HPLC-UVD) (Shimadzu CBM-20A, Tokyo, Japan) comprising a CBM-20A system controller, SIL-20A autosampler, SPD-M20A diode array detector, LC-20AD solvent delivery unit, DGU-20A3R degassing unit, and CTO-20A column oven. The extracts (20 μL) were separated on a Shim-pack ODS 5-µm column (Shimadzu, Kyoto, Japan) at 40 °C with a gradient solvent system consisting of water-acetonitrile (80:20 to 0:100 *v*/*v*) for 50 min. The flow rate was 1.0 mL/min, and the detection wavelength was 205 nm. The L-sulforaphane levels in each sample were quantified using a standard curve and expressed as μg/g of dry weight.

### 2.11. Statistical Analysis

All experiments were performed in triplicate. GraphPad Prism 7.0 software (La Jolla, CA, USA) was used for statistical analysis. Statistical analysis of TPC, TFC, and intracellular ROS generation was performed using one-way analysis of variance (ANOVA) with Tukey’s multiple comparison test (at 95% level of significance). Data were expressed as the mean ± standard deviation (SD), and statistical significance was identified at *p* < 0.05.

## 3. Results and Discussion

### 3.1. Total Polyphenol Content (TPC) and Total Flavonoid Content (TFC) of Broccoli Floret Extracts

Phenolics and flavonoids are large groups of plant secondary metabolites that are abundant in fruits and vegetables [16,17]. Phenolics and flavonoids are structurally and functionally diverse and have a wide range of biological activities, making them attractive ingredients for food technology research [18]. Notably, phenolics and flavonoids have gained the attention of many researchers in the field of food technology as potent antioxidants [18]. In broccoli, phenolics and flavonoids, including gallic acid, ellagic acid, salicylic acid, syringic acid, caffeic acid, ferulic acid, sinapic acid, isoquercetin, hyperoside, and rutin are found in differing quantities [19].

**Figure 2:**
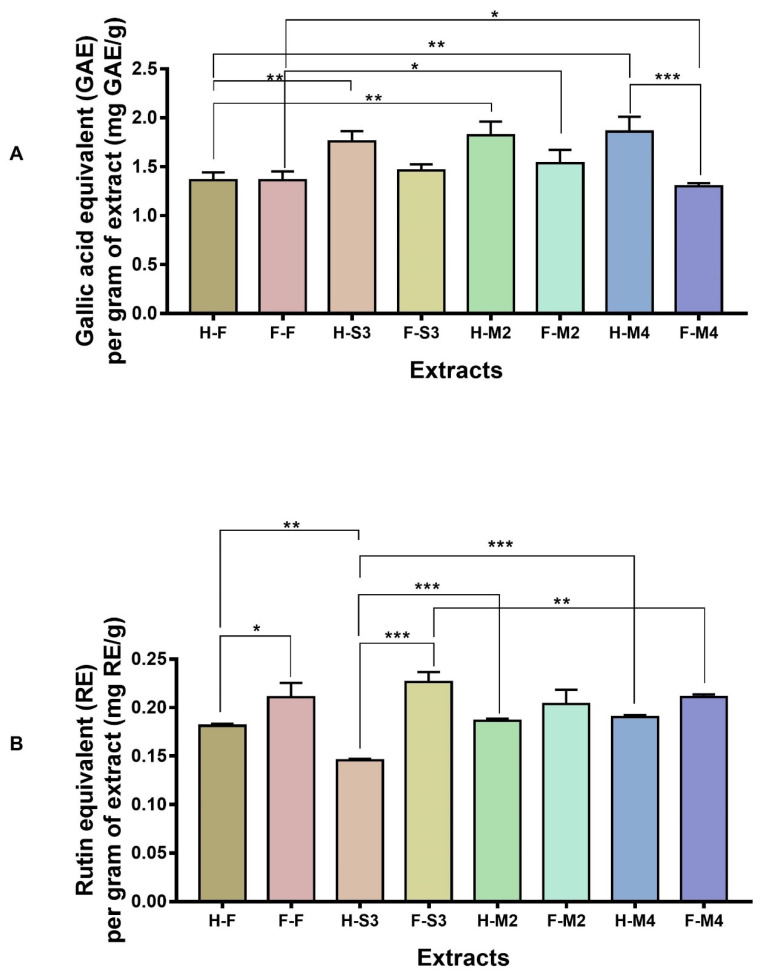
TPC (**A**) and TFC (**B**) of fresh-hot-air-dried (H-F), steamed (3 min)-hot-air-dried (H-S3), microwaved (2 min)-hot-air-dried (H-M2), microwaved (4 min)-hot-air-dried (H-M4), fresh-freeze-dried (F-F), steamed (3 min)-freeze-dried (F-S3), microwaved (2 min)-freeze-dried (F-M2), and microwaved (4 min)-freeze-dried (F-M4) samples. Statistical comparison among groups was carried out using one-way ANOVA with Tukey’s multiple comparison test. * *p* < 0.05, ** *p* < 0.01, and *** *p* < 0.001.

The TPCs of extracts from broccoli florets subjected to various cooking and processing conditions are shown in Figure 2A. First, we compared the TPC between fresh hot-air-dried (H-F) and fresh freeze-dried (F-F) samples. The extracts of F-F and H-F showed nearly equal TPC (Figure 2A). After comparing the TPCs of F-F and H-F, we compared the TPCs of H-S3, H-M2, and H-M4 with H-F to determine whether significant differences existed among the hot-air-dried groups: H-F vs. H-S3, H-F vs. H-M2, and H-F vs. H-M4. According to the TPC of extracts from hot-air-dried samples, H-S3, H-M2, and H-M4 contained higher TPC than the H-F sample, and this difference was significant (Figure 2A). TPCs of the extracts of H-F, H-S3, H-M2, and H-M4 were 1.36 ± 0.07, 1.75 ± 0.07, 1.82 ± 0.14, and 1.85 ± 0.15 mg GAE/g, respectively. Consistent with our findings, Turkmen et al. (2005) reported that heat treatment increased the TPC of broccoli by increasing the levels of free phenolics through disruption of inter-molecular interactions between phenolics [20]. Gliszczyńska-Świgło et al. (2006) demonstrated similar effects from steaming on the TPC of broccoli [21]. Şengül et al. (2014) showed that steaming can increase TPC in broccoli [22].

Then, the TPCs of the extracts of freeze-dried samples (F-S3, F-M2, and F-M4) were compared with the TPC of the F-F sample. F-S3 and F-M2 contained higher TPC than the F-F sample. The TPCs of freeze-dried samples F-F, F-S3, F-M2, and F-M4 were 1.36 ± 0.09, 1.46 ± 0.06, 1.53 ± 0.13, and 1.29 ± 0.03 mg GAE/g, respectively. Among these, the group comparison of F-F vs. F-M2 showed a significant increase in TPC (Figure 2A). A recent investigation reported elevated TPC in broccoli following microwave treatment [23]. The group comparison F-F vs. F-S3 also showed an increase in TPC, but this difference was not significant. TPC in the group F-F vs. F-M4 comparison showed a significant decrease (Figure 2A). In contrast, in the investigation by Şengül et al. (2014) [22], broccoli florets subjected to microwaving showed reduced TPC relative to raw samples, indicating that microwave can cause physical and chemical alterations to vegetables.

In addition, the TPCs of steamed hot-air-dried, steamed freeze-dried, microwaved hot-air-dried, and microwaved freeze-dried samples were grouped (H-S3 vs. F-S3, H-M2 vs. F-M2, H-M4 vs. F-M4, H-S3 vs. H-M2, H-S3 vs. H-M4, F-S3 vs. F-M2, and F-S3 vs. F-M4) and compared to determine whether hot-air-drying or freeze-drying had a significant effect on the TPC of cooked samples. Among these comparisons, the TPCs of H-S3 vs. F-S3, H-M2 vs. F-M2, and H-M4 vs. F-M4 showed an interesting pattern wherein freeze-dried samples (F-S3, F-M2, and F-M4) exhibited reduced TPC compared to hot-air-dried (H-S3, H-M2, and H-M4) samples (Figure 2A). Although the groups containing steamed hot-air-dried and microwaved hot-air-dried samples (H-S3 vs. H-M2 and H-S3 vs. H-M4) did not show a statistically significant difference in TPC, microwaved-hot-air-dried samples (H-M2 and H-M4) showed increased TPC compared with steamed-hot-air-dried samples (H-S3; Figure 2A). Similar to the H-S3 vs. H-M2 and H-S3 vs. H-M4 comparisons, TPCs of the steamed freeze-dried and microwaved freeze-dried groups (F-S3 vs. F-M2 and F-S3 vs. F-M4) showed no statistically significant difference (Figure 2A). However, F-M2 had a slightly higher TPC than F-S3, while F-M4 showed a slightly reduced TPC compared with F-S3 (Figure 2A).

In contrast to the TPC data, the TFCs of extracts of F-F and H-F samples showed differing patterns (Figure 2B). TFCs of H-F and F-F samples were first compared. The extract of F-F (0.21 ± 0.01 mg RE/g) had a significantly higher TFC than H-F (0.18 ± 0.001 mg RE/g) (Figure 2B). Then, we compared the TFCs of H-S3, H-M2, and H-M4 with the TFC of H-F according to the following groups: H-F vs. H-S3, H-F vs. H-M2, and H-F vs. H-M4. The group comparison of H-Fand H-S3 showed a significant relationship, with H-S3 having a lower TFC than the H-F sample (Figure 2B). Recently, Wu et al. (2019) reported that steam treatment can decrease the concentration of many kaempferol- and quercetin-derived flavonoids in broccoli [24]. H-M2 and H-M4 displayed slightly higher TFCs than H-F, with a non-significant relationship (Figure 2B). Similar to our results, Wu et al. (2019) also detected slight increases in flavonoid concentrations following microwave treatment [24]. The TFCs of the extracts from H-S3, H-M2, and H-M4 were 0.14 ± 0.001, 0.18 ± 0.002, and 0.19 ± 0.002 mg RE/g, respectively.

The total flavonoids of extracts of freeze-dried samples (F-S3, F-M2, and F-M4) were then compared with the total flavonoids of F-F sample. Freeze-dried samples exhibited total flavonoid content nearly equal to the total flavonoid content of the F-F sample (Figure 2B). The TFCs of freeze-dried samples F-S3, F-M2, and F-M4 were 0.22 ± 0.01, 0.2 ± 0.01, and 0.21 ± 0.002 mg RE/g, respectively. No significant difference in TFC was observed among the groups (Figure 2B). The TFCs of steamed hot-air-dried, steamed freeze-dried, microwaved hot-air-dried, and microwaved freeze-dried samples were grouped (H-S3 vs. F-S3, H-M2 vs. F-M2, H-M4 vs. F-M4, H-S3 vs. H-M2, H-S3 vs. H-M4, F-S3 vs. F-M2, and F-S3 vs. F-M4) and compared to determine whether hot-air-drying or freeze-drying had significant effects on the TFC of cooked samples. Among these comparisons, the TFCs of the groups H-S3 vs. F-S3, H-M2 vs. F-M2, and H-M4 vs. F-M4 displayed the opposite patterns to TPC, with freeze-dried samples (F-S3, F-M2, and F-M4) having higher TFC than hot-air-dried (H-S3, H-M2, and H-M4) samples (Figure 2B). The comparisons of H-S3 vs. H-M2 and H-S3 vs. H-M4, between steamed-hot-air-dried and microwaved-hot-air-dried samples, showed statistically significant differences in TFC, with microwaved hot-air-dried samples (H-M2 and H-M4) having elevated TFCs compared to the steamed hot-air-dried sample H-S3 (Figure 2B). Compared to F-S3, F-M2 and F-M4 showed reduced TFC (Figure 2B) and the TFC comparison of F-S3 vs. F-M4 showed a statistically significant difference (Figure 2B).

### 3.2. Antioxidant Activities

**Figure 3:**
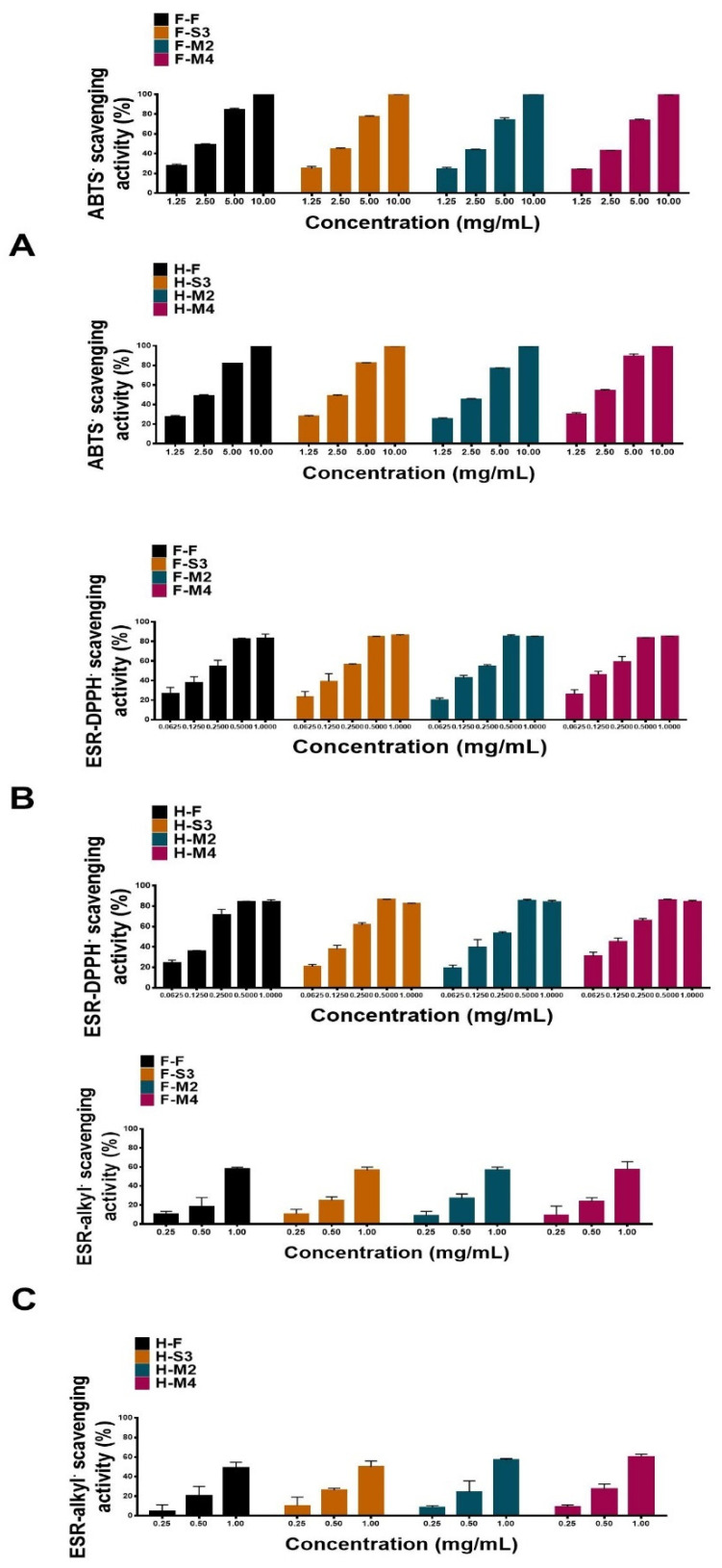
ABTS^•^ (**A**), DPPH^•^ (**B**), and alkyl^•^ (**C**) free radical scavenging potentials of broccoli extracts [fresh-hot-air-dried (H-F), steamed (3 min)-hot-air-dried (H-S3), microwaved (2 min)-hot-air-dried (H-M2), microwaved (4 min)-hot-air-dried (H-M4), fresh-freeze-dried (F-F), steamed (3 min)-freeze-dried (F-S3), microwaved (2 min)-freeze-dried (F-M2), and microwaved (4 min)-freeze-dried (F-M4)].

Several studies have investigated the effects of different cooking methods on the antioxidant activity of broccoli [20,21,22,23,24]. To obtain a comprehensive profile of the antioxidant activities of various broccoli extracts, we employed three antioxidant assessment methods, namely ABTS^•^, ESR-DPPH^•^, and ESR-alkyl^•^. These assays have been widely used to assess the free radical scavenging activities of various vegetables, fruits, individual compounds, and biological systems [14]. ABTS^•^ is a colored molecule that can be reduced to colorless ABTS when mixed with any oxidizable agent [25]. Compared to colorimetric methods, ESR spectrometry is a sensitive technique used to assess free radicals or transition metal ions [26]. As shown in Figure 3A–C, the ABTS^•^, ESR-DPPH^•^, and ESR-alkyl^•^ scavenging activities of broccoli extracts (F-F, F-S3, F-M2, F-M4, H-F, H-S3, H-M2, and H-M4) increased with increasing concentration. All broccoli extracts displayed greater than 70% ABTS^•^ and ESR-DPPH^•^ radical scavenging activities at the highest doses tested (10 and 1 mg/mL, respectively) (Figure 3A,B). Similar to the results for ABTS^•^ and ESR-DPPH^•^ radical scavenging activities, all broccoli extracts also demonstrated ESR-alkyl^•^ scavenging activities in a dose-dependent manner, with more than 50% inhibition observed at the highest extract doses (Figure 3C). The EC_50_ values obtained from each antioxidant assay are listed in Table 1. From these EC_50_ values, it is apparent that the H-M4 extract had strong radical scavenging effects. As shown in Figure 2A, a significant increase in TPC, one of the major contributors to antioxidant activity, may contribute to the enhanced antioxidant activity of H-M4. All other extracts displayed similar

EC_50_ values (Table 1), suggesting that the tested cooking and processing techniques did not significantly alter the antioxidant activities of broccoli florets. The EC_50_ values obtained for positive controls catechin in ESR-DPPH^•^ and ESR-alkyl^•^ and α-tocopherol in ABTS^•^ assays were 2.83 µM, 11.85 µM and 19.53 µM, respectively. Although all broccoli extracts showed antioxidant potentials in the ABTS^•^, ESR-DPPH^•^, and ESR-alkyl^•^ assays, these activities did not correlate with the TPC and TFC values. The reason for the highly similar antioxidant potentials (based on EC_50_ values) (Table 1) among broccoli extracts may be related to the presence of differing quantities of secondary metabolites other than phenolics and flavonoids, which exert stronger antioxidant potentials that mask the effects of phenolics and flavonoids. Therefore, identification and quantification of other secondary metabolites are warranted to obtain a comprehensive picture of the antioxidant potentials and associated secondary metabolites in broccoli extracts. Glucosinolates and isothiocyanates have been reported to contribute strongly to the antioxidant potential of broccoli extracts [4].

### 3.3. H_2_O_2_-Induced ROS Production

**Figure 4:**
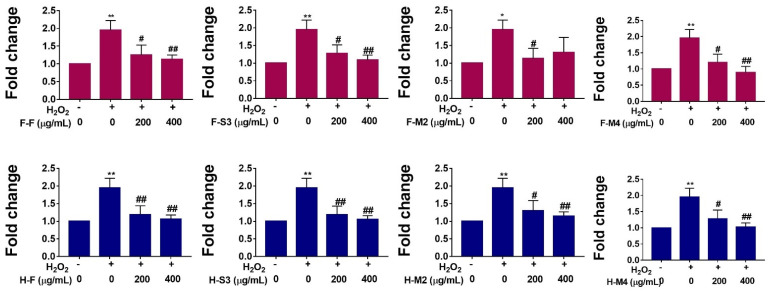
Broccoli extracts (fresh-hot-air-dried (H-F), steamed (3 min)-hot-air-dried (H-S3), microwaved (2 min)-hot-air-dried (H-M2), microwaved (4 min)-hot-air-dried (H-M4), fresh-freeze-dried (F-F), steamed (3 min)-freeze-dried (F-S3), microwaved (2 min)-freeze-dried (F-M2), and microwaved (4 min)-freeze-dried (F-M4)) inhibit H_2_O_2_-induced reactive oxygen species (ROS) production. HepG2 cells were pretreated with 200 or 400 µg/mL broccoli extracts prior to exposure to 300 µM H_2_O_2_ for 10 min. 2′,7-dichlorodihydrofluorescein (H_2_DCFDA) diacetate was used as a probe to measure intracellular ROS levels. Data are expressed as mean ± standard deviation of three independent experiments. * *p* < 0.05 and ** p < 0.01 vs. untreated control group; ^#^ *p* < 0.05 and ^##^ *p* < 0.01 vs. H_2_O_2_ only treated group.

Oxidative stress, an imbalance in the production and elimination of ROS, has been reported to play key roles in the pathogenesis of various liver disorders, including liver cancer [27]. H_2_O_2_ is one of the key causes of oxidative injury, as it can be easily transformed into hydroxyl radicals, which are considered destructive free radicals [14]. H_2_O_2_ has been used as an inducer of ROS-mediated oxidative stress in several in vitro models [14]. Moreover, a number of plant extracts and natural compounds have been reported to reduce H_2_O_2_-induced ROS production based on in vitro studies [14]. Here, we examined the effects of broccoli extracts on ROS generation in HepG2 cells exposed to H_2_O_2_ (300 µM). As indicated in Figure 4, H_2_O_2_ treatment significantly increased ROS production, with an almost two-fold increase relative to the untreated controls. However, intracellular ROS generation was significantly decreased in the experimental groups pre-treated with broccoli extracts compared to the H_2_O_2_-induced groups (Figure 4). Among these experimental groups, H-S3, H-M4, F-S3, and F-M4 showed greater reductions in intracellular ROS generation than other groups (Figure 4). These observations suggest that H_2_O_2_ can induce ROS generation in HepG2 cells and the extracts of cooked broccoli can effectively reduce H_2_O_2_-induced ROS production (Figure 4). Studies assessing the effects of broccoli extracts on H_2_O_2_-induced ROS production are extremely limited. Park et al. (2014) reported cytoprotective effects of broccoli extracts in PC12 cells following exposure to H_2_O_2_ [28]. Moreover, a recent study demonstrated that sulforaphane, which is found in broccoli, could effectively attenuate H_2_O_2_-induced oxidative stress in human osteoarthritic chondrocytes [29]. Another recent study reported that sulforaphane can attenuate H_2_O_2_-induced oxidative stress in vitro [30].

### 3.4. Effects of Broccoli Extracts on Cell Viability

**Figure 5:**
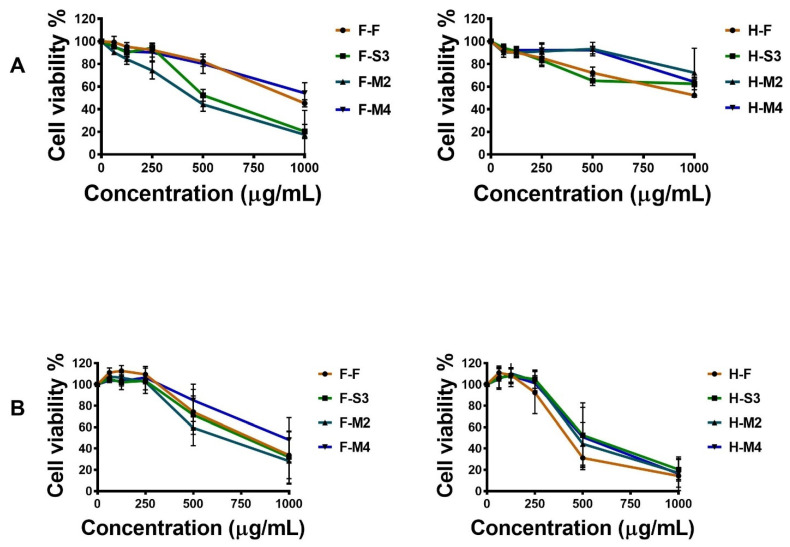

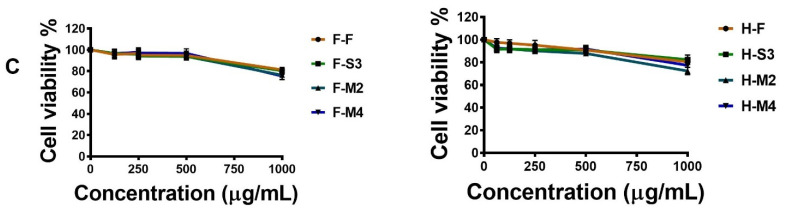
Cytotoxic effects of broccoli extracts (fresh-hot-air-dried (H-F), steamed (3 min)-hot-air-dried (H-S3), microwaved (2 min)-hot-air-dried (H-M2), microwaved (4 min)-hot-air-dried (H-M4), fresh-freeze-dried (F-F), steamed (3 min)-freeze-dried (F-S3), microwaved (2 min)-freeze-dried (F-M2), and microwaved (4 min)-freeze-dried (F-M4)) in MCF-7 (**A**), MDA-MB-231 (**B**), and MCF-10A (**C**) cells as assessed using the MTT assay.

Uncontrolled cell proliferation and evasion of apoptosis are hallmark features of cancer cells [31]. The anti-proliferative effects of broccoli extracts in MCF-7 breast cancer cells, MDA-MB-231 triple-negative breast cancer cells, and MCF-10A normal mammary epithelial cells were assessed using the MTT assay following 48 h of exposure to broccoli extracts (Figure 5). We observed that cell proliferation was inhibited in breast cancer cells in a dose-dependent manner following exposure to the broccoli extracts (Figure 5). F-S3 and F-M2 showed greater anti-proliferative activities in MCF-7 cells (Figure 5A), while F-M2, H-F, H-M2, and H-M4 showed greater anti-proliferative effects in MDA-MB-231 cells (Figure 5B). The F-F, F-M4, H-F, H-S3, H-M2, and H-M4 extracts exhibited higher IC_50_ values in MCF-7 cells, whereas F-F, F-S3, and F-M4 had higher IC_50_ levels in MDA-MB-231 cells (Table 2). These results demonstrate that broccoli extracts can inhibit the proliferation of breast cancer cells to varying degrees. Interestingly, broccoli extracts exhibited few cytotoxic effects in normal mammary epithelial cells at the same doses (Table 2, Figure 5C). A recent study by Le at al. (2019) reported in vitro anti-proliferative effects of extracts of broccoli sprouts in lung (A549), liver (HepG2), and colon (Caco-2) cancer cells. Extracts of broccoli sprouts had low cytotoxicity to FL83B normal mouse liver cells [32]. Furthermore, broccoli and collard extracts have been reported to show cytotoxic effects in MCF-7 breast cancer cells at concentrations of 50 and 100 mg/mL [33]. According to the results of cell proliferation assays, we were unable to find a positive correlation between TPC or TFC and anti-proliferative activities of broccoli extracts. However, the F-S3 and F-M2 extracts, which had high concentrations of L-sulforaphane, exhibited strong cytotoxic effects in MCF-7 breast cancer cells, suggesting a possible relationship between the L-sulforaphane content and cytotoxic activity.

### 3.5. L-Sulforaphane Quantification in Broccoli Extracts Using HPLC

Members of the plant family Brassicaceae are rich in secondary metabolites, including glucosinolates and various isothiocyanates [3,4]. The enzyme myrosinase, in the glycoside hydrolase family, converts glucosinolates into the corresponding sulforaphanes [3,4]. Various cooking and processing techniques have been reported to affect the conversion rate of glucosinolates into sulforaphanes via enzyme hydrolysis. L-sulforaphane is the biologically active isomer, and has been reported to exert strong antioxidant, anti-inflammatory, anti-cancer, and chemopreventive effects [34]. Considering the importance of L-sulforaphane as an emerging antioxidant and anti-cancer agent, we quantified L-sulforaphane contents in the broccoli extracts using HPLC. Increased L-sulforaphane concentrations were observed in the F-S3 and F-M2 extracts compared with F-F (Table 3). Hot-air-dried samples exhibited lower L-sulforaphane contents than freeze-dried samples. Among hot-air-dried samples, L-sulforaphane concentrations were reduced relative to H-F by steam or microwave treatment (Table 3).

## 4. Conclusions

In general, steam and microwave treatments increased the TPC of broccoli florets. TFC was reduced following steam treatment, while a slight increase in TFC was observed in microwaved samples. The H-M4 extract exerted stronger radical scavenging effects than other extracts. Furthermore, the H-S3, H-M4, F-S3, and F-M4 extracts showed strong reductions in H_2_O_2_-induced ROS generation. HPLC quantification demonstrated that the F-S3 and F-M2 extracts exhibited elevated levels of L-sulforaphane. In addition, those two extracts showed strong anti-proliferative effects in MCF-7 breast cancer cells.

Although several studies have reported that antioxidant activity is lost during cooking, we did not observe a significant decrease in the antioxidant activity of broccoli following steam and microwave treatments. Therefore, to fully elucidate the antioxidant properties of broccoli after various cooking methods, more research is needed, preferably using cooking and processing methods other than steam and microwave, as well as different extraction methods to isolate total phenolics and flavonoids. As we observed no significant decline in the antioxidant activity of broccoli florets, the cooking and processing methods and conditions used in the present investigation are recommended.

## Figures and Tables

**Table 1:** EC_50_ (mg/mL) values obtained from various antioxidant assays for broccoli extracts (H-F, H-S3, H-M2, H-M4, F-F, F-S3, F-M2, and F-M4).

	ABTS^•^	ESR-DPPH^•^	ESR-alkyl^•^
H-F	1.98	0.15	1.49
H-S3	1.97	0.17	1.26
H-M2	2.23	0.18	1.17
H-M4	1.67	0.13	1.03
F-F	1.92	0.17	1.22
F-S3	2.23	0.16	1.12
F-M2	2.35	0.17	1.11
F-M4	2.39	0.15	1.18

**Table 2:** IC_50_ values (µg/mL) of broccoli extracts (F-F, F-S3, F-M2, F-M4, H-F, H-S3, H-M2, and H-M4) in MCF-7 and MDA-MB-231 cells following 48-h exposure as assessed using the MTT assay.

Breast Cancer Cells	F-F	F-S3	F-M2	F-M4	H-F	H-S3	H-M2	H-M4
MCF-7	1381 ^a^ ± 10.590	651 ^b^ ± 17.030	447 ^c^ ± 4.500	1505.3 ^d^ ± 3.210	1195.3 ^e^ ± 54.590	1295 ^f^ ± 55.360	2988 ^g^ ± 10.260	2365 ^h^ ± 15.090
MDA-MB-231	1355 ^a^ ± 12.500	1130 ^b^ ± 7.780	948 ^c^ ± 12.100	1846 ^d^ ± 4.200	570 ^e^ ± 11.410	820 ^f^ ± 15.140	723 ^g^ ± 13.410	747 ^h^ ± 15.470
MCF-10A	>1000	>1000	>1000	>1000	>1000	>1000	>1000	>1000

Means with the different letters (a–h) are significantly different in a row at a 95% confidence interval.

**Table 3:** Quantification of L-sulforaphane in broccoli extracts (F-F, F-S3, F-M2, F-M4, H-F, H-S3, H-M2, and H-M4) using HPLC.

Component	F-F	F-S3	F-M2	F-M4	H-F	H-S3	H-M2	H-M4
L-sulforaphane	0.11 ^a^ ± 0.001	0.18 ^b^ ± 0.001	0.38 ^c^ ± 0.004	0.003 ^d,i^ ± 6.6 × 10^−5^	0.074 ^e^ ± 0.001	0.011 ^f^ ± 0.0003	0.005 ^g,i^ ± 0.0003	0.002 ^h,i^ ± 3.83 × 10^−6^

Data are expressed as µg/g of extract from three independent quantifications. Means with the same letter are not significantly different in a row at 95% confidence interval.

## Data Availability

Not applicable.
